# Research Review on Quality Detection of Fresh Tea Leaves Based on Spectral Technology

**DOI:** 10.3390/foods13010025

**Published:** 2023-12-20

**Authors:** Ting Tang, Qing Luo, Liu Yang, Changlun Gao, Caijin Ling, Weibin Wu

**Affiliations:** 1 College of Engineering, South China Agricultural University, Guangzhou 510642, China; 20203163056@stu.scau.edu.cn (T.T.); luoyq@scau.edu.cn (Q.L.); yl1009496755@163.com (L.Y.); gao666@stu.scau.edu.cn (C.G.); 2 Tea Research Institute, Guangdong Academy of Agricultural Sciences, Guangzhou 510640, China

**Keywords:** fresh tea leaves, hyperspectral imaging technology, spectroscopy, analytic method

## Abstract

As the raw material for tea making, the quality of tea leaves directly affects the quality of finished tea. The quality of fresh tea leaves is mainly assessed by manual judgment or physical and chemical testing of the content of internal components. Physical and chemical methods are more mature, and the test results are more accurate and objective, but traditional chemical methods for measuring the biochemical indexes of tea leaves are time-consuming, labor-costly, complicated, and destructive. With the rapid development of imaging and spectroscopic technology, spectroscopic technology as an emerging technology has been widely used in rapid non-destructive testing of the quality and safety of agricultural products. Due to the existence of spectral information with a low signal-to-noise ratio, high information redundancy, and strong autocorrelation, scholars have conducted a series of studies on spectral data preprocessing. The correlation between spectral data and target data is improved by smoothing noise reduction, correction, extraction of feature bands, and so on, to construct a stable, highly accurate estimation or discrimination model with strong generalization ability. There have been more research papers published on spectroscopic techniques to detect the quality of tea fresh leaves. This study summarizes the principles, analytical methods, and applications of Hyperspectral imaging (HSI) in the nondestructive testing of the quality and safety of fresh tea leaves for the purpose of tracking the latest research advances at home and abroad. At the same time, the principles and applications of other spectroscopic techniques including Near-infrared spectroscopy (NIRS), Mid-infrared spectroscopy (MIRS), Raman spectroscopy (RS), and other spectroscopic techniques for non-destructive testing of quality and safety of fresh tea leaves are also briefly introduced. Finally, in terms of technical obstacles and practical applications, the challenges and development trends of spectral analysis technology in the nondestructive assessment of tea leaf quality are examined.

## 1. Introduction

The tea tree belongs to the tea group of plants in the genus Camellias of the family Camelliaceae. Tea tree is an important economic crop. Especially for the current stage of China, the tea industry is an important treasure to promote China’s agricultural economic development and rural revitalization. China has a long history of tea culture and is a large country in terms of plantation production and consumption. According to the statistics of the China Tea Circulation Association, from 2011 to 2022, the area of tea plantation, the total annual output of dry gross tea, and the total annual output value of dry gross tea have increased by 157.6%, 196.0%, and 404.2%, respectively [1]. There are more than 700 known chemical components in tea. These include primary metabolites of proteins, sugars, fats, and secondary metabolites in the tea tree—polyphenols, pigments, theanines, alkaloids, aromatic substances, and saponins. They not only affect the formation of tea color, aroma, and flavor but also play an important role in the nutritional and health effects of tea [2]. Tea’s main uses include waking up, sleeping, relieving fever, aiding in digestion, decreasing gas, expectoration, treating fistulas, facilitating urination, facilitating the large intestine, decreasing miasma, clearing the head and eyes, helping with dysentery, facilitating the small intestine, decreasing headaches, sores, stroke, and sunstroke, aiding in sobriety, and so on [3]. Often used as an herbal remedy throughout history, tea has evolved into a popular beverage that has tremendous economic, health, and cultural value in the marketplace. With the spread and development of tea culture, consumers are demanding more and more regarding the quality of tea. Nowadays, the quality of tea is mainly assessed by sensory review, physical and chemical testing, and emerging technological testing [4].

The sensory quality of tea refers to the comprehensive effect of the many compounds in tea, especially the substances that can be dissolved in tea broth, on the sensory stimulation of the human body. It is mainly composed of appearance, color, aroma, taste, and other factors. Shape and color are the external factors of tea quality, while aroma and taste are the internal core quality factors of tea. The evaluation of tea quality through the sensory review method requires the reviewer to undergo a long period of training and a lot of experience. In addition, the review results are subject to a review of the environment, individual sensory sensitivity differences, and other factors of interference and influence, resulting in the review of the results possessing strong subjectivity. Physical testing techniques mainly include the use of an electronic balance and oven to determine the quality and moisture content of tea leaves. The observation and analysis of the phenotype and structure of tea leaves have been carried out using a microscope [5,6]. Conventional chemical detection techniques mainly include High-Performance Liquid Chromatography (HPLC), Gas Chromatography (GC), Mass Spectrometry (MS), Gas Chromatography-Mass Spectrometry (GC-MS), and the titrimetric method [7]. They are diagnostic analytical methods to detect the content of compounds in tea at the molecular level. These are usually used in combination with emerging techniques such as HSI, MIRS, RS, NIRS, and other scientific techniques. Physicochemical testing techniques are more mature, with more accurate and objective results, which are necessary for the quantitative evaluation of tea quality. However, traditional chemical methods need to be coupled with chemical reagents to titrate the reaction or need to be observed and analyzed with the aid of chromatographic instruments to analyze tea broth preparation after extraction and separation [8]. This method of measuring plant biochemical indicators is time-consuming, labor-costly, and complicated to operate [9]. As a result, the realization and development of tea quality monitoring has been severely constrained. In recent years, researchers have been exploring fast and accurate techniques to monitor tea quality. RGB imaging, multispectral imaging, HSI, nuclear magnetic resonance imaging (NMRI), NIRS, RS, electronic noses, electronic tongues, etc. are often applied in emerging technologies to realize non-destructive and rapid detection of tea quality.

As the raw material for tea production, the quality of tea leaves directly affects the quality of finished tea. The ratios of polyphenols to amino acids, polysaccharides, and caffeine content of tea leaves are one of the most important factors affecting the aroma, nutrition, and color of finished tea, while the fiber content determines the tenderness of tea leaves [10]. Non-destructive monitoring of the quality and material content of fresh tea leaves in situ can not only accurately grasp the growth of the tea tree but also assist in the decision-making process of tea-picking programs to ensure the quality of tea leaves [11]. Spectroscopic detection technology is widely used in rapid non-destructive testing of the quality and safety of agricultural products due to its advantages of rapidity, accuracy, and on-line real-time detection [12,13,14]. Spectral analysis is a qualitative and quantitative analysis of the composition of a sample using the unique absorption or emission spectral features of different substances in different spectral ranges. Due to its advantages of rapid, non-destructive, multiple simultaneous testing, and portability, spectral analysis finds wide applications in the quality testing of fresh tea leaves. At present, the most commonly used spectral analysis methods include HSI, NIRS, MIRS, Terahertz spectroscopy (THz), RS, and Fluorescence spectroscopy (FS).

NIRS obtains information by measuring the absorption and reflection of Near-infrared (NIR) light from a sample. NIR light is absorbed in the frequency band associated with molecular vibrations and chemical bonding and, therefore, provides information about the composition of the sample. MIRS focuses on the mid-infrared band and provides information on molecular vibrations and the rotation of matter. Different molecules and the bonds between them are uniquely characterized in the mid-infrared spectrum. The THz band is located between the microwave and infrared bands, which is highly penetrating. This is suitable for studying crystal structures, plant cell walls, moisture, and more. RS provides information about molecular vibrations and rotations based on the frequency shift of the light that is scattered from the sample. It has both high sensitivity and resolution. FS is based on the fluorescence signal emitted by the sample when exposed to excited light and is used to analyze fluorescently active substances. It is sensitive to biomolecules and pigments. These spectroscopic techniques help in the study of the chemical structure of fresh tea leaves’ moisture, aroma composition, and pigment composition determination. Although these spectroscopic techniques have a wide range of applications in tea research, HSI is able to provide both rich spectral information and high-resolution spatial information. This grants HSI unique advantages in tea research in terms of quality assessment, authenticity identification, and growth environment monitoring. In addition, hyperspectral reflectance data, mid-infrared spectral data, Raman data, and terahertz data are all acquired by spectral techniques, and generally speaking, the steps of their data processing all include noise reduction, dimensionality reduction, feature extraction, and modeling. The data analysis of HSI includes image information analysis in addition to spectral information analysis. Therefore, in this paper, in order to keep readers abreast of the latest spectral technology at home and abroad in tea fresh leaves and the research and application progress, through China’s knowledge network and the Web of Science literature database, this study employs the key words tea fresh leaves and spectral collation to review the last ten years of relevant literature. This study focuses on the principle of HSI technology, the analysis method, and its application in the non-destructive evaluation of the quality and safety of fresh tea leaves. At the same time, the principles and applications of other spectroscopic techniques are briefly introduced, including the application of MIRS, NIRS, RS, and other spectroscopic techniques in the nondestructive testing of the quality and safety of fresh tea leaves. Finally, the challenges and development trends of spectral analysis techniques in nondestructive testing of tea quality are discussed in terms of technical difficulties and practical applications.

## 2. Spectral Technology

### 2.1. Hyperspectral Imaging Technology

**Figure 1:**
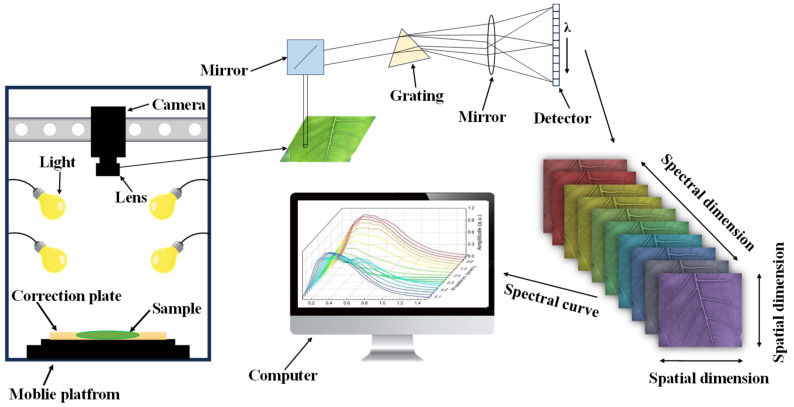
HSI working principle diagram.

HSI is a combination of spectral detection technology and image technology. The difference between active and passive hyperspectral techniques is whether an active light source is required. Depending on the hyperspectral imaging method, the active hyperspectral imaging system is divided into four categories, namely swing-sweep, push-sweep, condensed acquisition, and snapshot [15]. The core devices of active hyperspectral imaging systems are generally light sources, spectroscopic elements, detectors, and data acquisition and processing systems [16]. Its working principle diagram is shown in Figure 1. The light source is an important part of an active hyperspectral imaging spectroscopy system. The three commonly used light sources are tungsten halogen lamps, quantum cascade lasers, and light-emitting diodes. The spectroscopic elements are mainly diffraction gratings and tunable filters. Detectors are key devices for converting optical signals into electrical signals in hyperspectral imaging systems. Currently, there are two main types of detectors used in hyperspectral imaging systems, namely line array detectors and surface array detectors. Optical signals can be converted into analog current signals, which are amplified, and the modulus to digital conversion of the current signals is used to acquire images. Data acquisition and processing systems are used to acquire spectral images collected from the camera and process and analyze these images.

While imaging the spatial features of the analyzed target, each spatial pixel is dispersed into dozens or even hundreds of narrow bands to achieve continuous spectral coverage [17]. Spectroscopic detection techniques utilize a series of spectral bands in a narrow wavelength range to capture spectral information reflected or emitted by an object. These bands typically include wavelengths in the visible, infrared, and ultraviolet ranges. Each band captures a different spectral signature of the object, thus providing detailed spectral data. Hyperspectral images are acquired through the use of hyperspectral cameras or sensors. These devices are capable of capturing images in a variety of wavelength ranges, often including hundreds to thousands of spectral channels [18]. Due to its benefits of high-spectrum resolution and the capacity to offer image and spectral information, HSI has steadily become a research hotspot and has been employed in a wide range of applications, including the quality inspection of fresh tea leaves. It mainly includes analyzing the chemical composition of tea, identifying the type and origin of tea, and detecting impurities.

### 2.2. Other Spectroscopic Technologies

**Figure 2:**
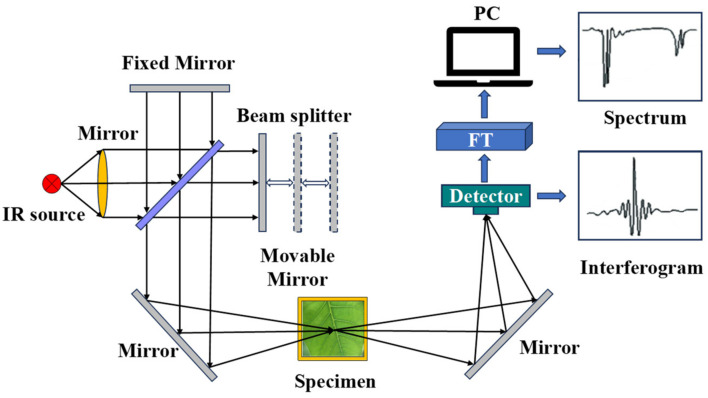
Infrared spectrometer working principle diagram.

**Figure 3:**
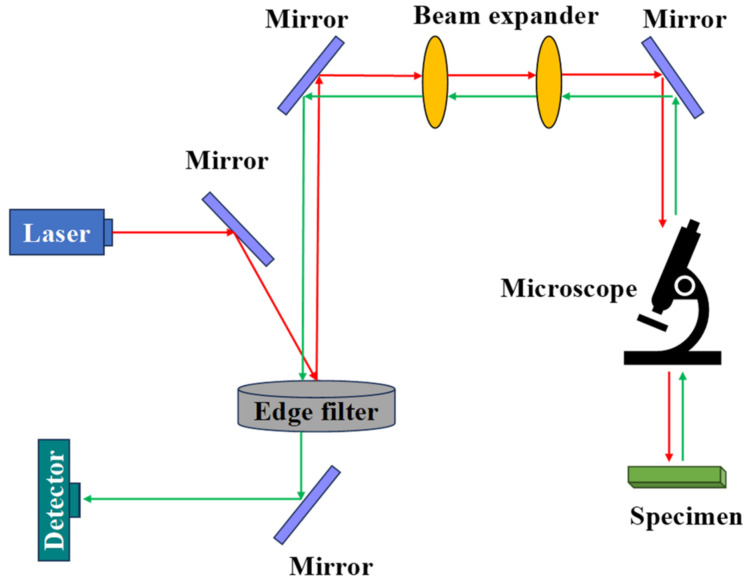
RS working principle diagram.

The NIRS and MIRS components mainly include an optical system, a detector, signal acquisition, and a processing module [1]. The working principle of the infrared spectroscopy system is shown in Figure 2. NIRS is a technique used to determine which functional groups are contained in a molecule based on the characteristic frequencies of the infrared absorption spectra, thus identifying unknown classes of compounds for qualitative analysis [19]. MIRS is composed of molecules with vibrational fundamental frequencies, multiple and broad absorption bands, high absorption intensities, and significant absorption characteristics that provide more information about frequencies and intensities. Most of the characteristic vibrational peaks of typical functional groups are distributed in the mid-infrared region [2]. Compared with NIRS, it has the advantages of relatively easy modeling and stable results. The in situ RS test system mainly consists of a Raman spectrometer, a Raman optical system, and a sample detection chamber [3]. The working schematic of the RS system is shown in Figure 3. RS and infrared spectroscopy are complementary to each other. Infrared spectroscopy is suitable for studying the polar bonding vibrations of different atoms, while RS is suitable for studying the non-polar bonding vibrations of the same atom [20].

**Figure 4:**
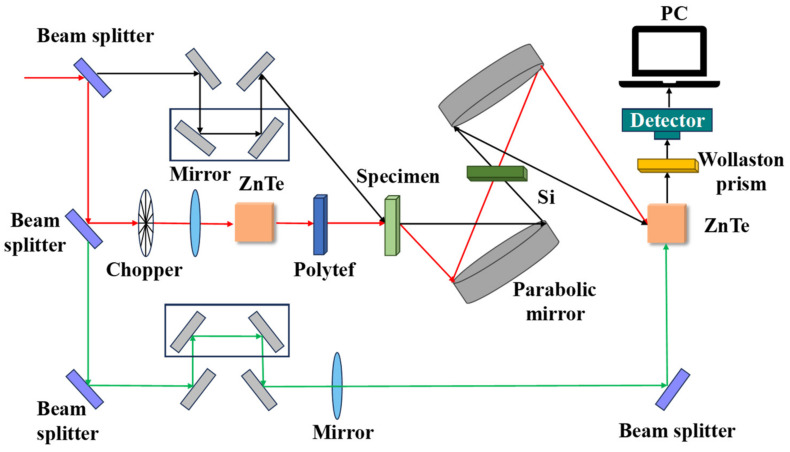
THz working principle diagram.

**Figure 5:**
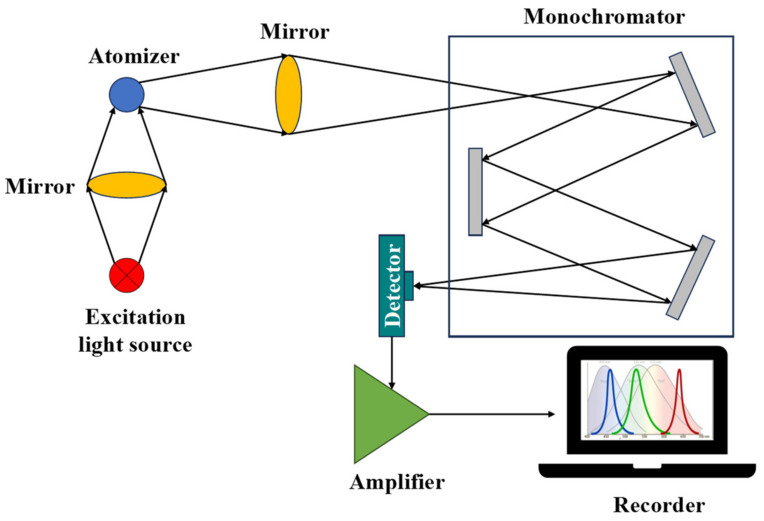
FS working principle diagram.

The THz system consists of a dual-laser-controlled intelligent electronic device, two distributed feedback lasers, and two fast scanning modes [4]. Its working schematic is shown in Figure 4. THz evaluates terahertz light using absorption, reflection, transmission, and other properties of a substance, which can be used for qualitative analysis of compounds [21]. The principle is to analyze the components of a mixture in the THz by using the absorption and transmission properties of a substance based on its absorption spectrum, refraction spectrum, dielectric coefficient, and other properties. The FS system consists of an excitation light source and a spectrometer [7]. Its working schematic is shown in Figure 5. FS is a method of quantitative and qualitative substance analysis based on the phenomenon of photoluminescence of substances and the investigation of fluorescence characteristics and intensity. [22]. Fluorescent compounds with different structures have unique excitation and emission spectra. Therefore, the shapes and peak positions of the excitation and emission spectra of fluorescent substances can be compared with the spectrograms of standard solutions for qualitative analysis. At low concentrations, the fluorescence intensity of a solution is proportional to the concentration of the fluorescent substance: F = Kc, where F is the fluorescence intensity, c is the concentration of the fluorescent substance, and K is the scale factor, which is the basis for the quantitative analysis of fluorescence spectra [23].

In Table 1, the advantages and disadvantages of several spectroscopic techniques are compared. In the analysis of tea fresh leaves, these spectroscopic techniques can be applied to study pigments, antioxidant substances, functional components, aroma substances, and the molecular structure of tea.

## 3. Hyperspectral Information Analysis Method for Tea Fresh Leaf Quality Testing

Hyperspectral information includes one-dimensional spectral information and two-dimensional spatial (image) information [34]. Spectral information can reflect the internal structure of the sample such as the molecular composition and can be applied for the quantitative and qualitative analysis of tea fresh leaves. Image information can reflect the external quality characteristics such as size, shape, and defects of the sample, which can be made use of for a qualitative examination of tea fresh leaves. The fusion of spectral and image technologies can not only study the internal composition content of the analyzed object but also visualize and analyze its distribution, which can be employed to capture the spectral information and spatial distribution of the target object.

### 3.1. Spectral Information Analysis

Raw spectral data usually need to undergo some pre-processing and analysis before they can be used for specific research or applications. The main reason for this is that its acquisition may be affected by a variety of interfering factors such as noise, baseline drift, light scattering, etc. [35]. Therefore, the data need to be processed for noise reduction as well as baseline correction. In addition, the raw data may contain a large amount of redundant information or unnecessary details, and the key information needs to be extracted using the feature band selection method. Furthermore, the analysis phase requires modeling according to the research objectives in order to obtain the required information or conclusions from the data. The steps of spectral data parsing include data preprocessing, feature band extraction, modeling, and model evaluation.

#### 3.1.1. Spectral Data Preprocessing

Spectral data preprocessing is a key step before analyzing spectral data, aiming at eliminating interference and improving data quality for subsequent analysis. Spectral data preprocessing mainly includes normalization, baseline correction, and noise reduction. The normalization method balances the distribution of variables and mean values by scaling the components of the data to a relatively consistent scale, which can attenuate the influence of factors such as light-range variation and sample sparsity on spectral information [36]. The normalization methods are Max-Min Normalization (MMN) and Vector Normalization (VN) [37,38]. MMN is a linear mapping of data to a specified range, usually [0, 1]. This process involves two key values: minimum (min) and maximum (max). By linearly transforming the data points, the min value is mapped to 0, the max value to 1, and the values in between will be distributed equiprimordially over this range. VN, on the other hand, distinguishes MMN, which, instead of mapping the data to a specific range, normalizes the data by changing its magnitude and direction. Its goal is to map data points to unit vectors.

Baseline correction is mainly used to correct the baseline shift problem in spectroscopy due to measurement variations of spectroscopic instruments or changes in measurement environment parameters [39]. Baseline correction methods include multiple scattering correction (MSC), standard normal variation (SNV), detrending (DT), orthogonal signal correction (OSC), and moving average (MA) [40,41,42,43,44]. The MSC method is used to correct the baseline translation and offset phenomena of spectral data by ideal spectra, which can effectively eliminate the scattering phenomena generated by uneven particle distribution and particle size, thus enhancing the correlation between spectra and data [45]. Similar to MSC, the SNV can also be used to correct the spectral errors caused by scattering between samples, but the algorithms are different. SNV is the process of subtracting the spectral value of each sample from the mean of the spectral value of that sample and dividing it by the standard deviation of the spectral value of that sample. This makes the processed spectral data conform to the standard normal distribution. It is mainly employed to eliminate the effects of diffuse reflections due to solid particle size, surface scattering, and variations in optical range [46]. Moreover, OSC is also used to eliminate errors arising from the surface scattering and baseline drift of spectral signals [47]. OSC is used to remove the information in the spectral matrix that is not related to the components to be measured by orthogonal projection and then carry out multivariate correction calculation. After achieving the purpose of simplifying the model, it then improves the predictive ability of the model [48]. MA is used to take the average of the data in a certain time period and use this average to represent the data in that time period, thus achieving the purpose of smoothing the data [49]. Spectral data contain information about the sample, but there may be some unrelated underlying trends in the data. These trends can be long-term variations in the data, usually related to time. They can also be trends due to other factors, such as temperature changes, instrument drift, etc. DT removes the trend or drift from the data [50]. DT usually involves fitting a trend model. Examples include linear regression or polynomial fitting, and then subtracting the estimates of this model from the raw data to obtain corrected data [51].

Noise reduction processing is performed by using various signal processing techniques and mathematical algorithms in order to remove or reduce the noise and retain the useful signal. Some of the methods for noise reduction are Savitzky–Golay smoothing (SG), first-order derivative (FD), second-order derivative (SD), Fourier Transform (FT), and Wavelet Transform (WT) [52,53,54]. SG smoothing reduces noise by smoothing the signal using a polynomial fit within a sliding window [55]. In addition, different window sizes and numbers of polynomials can be selected to balance the smoothing and noise suppression effects according to practical needs. The adaptability of SG smoothing methods to noise suppression and smoothing operations has led to their widespread use in spectral analysis. SG smoothing is often combined with FD and second-order derivatives for noise reduction in raw spectral data. The FD is the rate of change of the original signal and represents the slope or gradient in the signal. By calculating the FD, rapid changes or edges in the signal can be highlighted, thus helping to detect features and boundaries in the signal. The FD can help reduce high-frequency noise in a signal. The SD is the rate of change of the FD, which indicates the curvature in the signal. Calculating the SD helps to highlight features in the signal more strongly, especially spikes or troughs in the signal. This helps in identifying extreme points in the signal. SD can further reduce high-frequency noise and provide clearer information about features. The FT converts a signal from the time domain to the frequency domain, thereby breaking the signal into components of different frequencies. High- or low-frequency components can be selectively filtered out to extract the signal components of interest and reduce the effect of noise. Compared to FT, WT is a more flexible tool for signal analysis, as it is capable of local and multi-scale analysis of spectral data. The WT is used to effectively reduce noise and improve the signal-to-noise ratio of spectral data by decomposing the signal into wavelet functions at different scales and by analyzing and processing the different frequency components of the signal while retaining useful feature information. This makes spectral data easier to interpret and utilize. In Table 2, the characteristics and advantages and disadvantages of various pretreatment methods are summarized.

#### 3.1.2. Characteristic Band Screening

Since raw data may contain a lot of irrelevant information, feature band selection can help identify and enhance task-relevant information [56]. This helps to improve the interpretability of the data. Also, feature band selection can reduce the dimensionality of the data, thus reducing the cost of data storage and processing. The selection of representative feature bands can reduce the size of the dataset without losing important information. The methods for feature band selection are stepwise discriminant analysis (SDA), the successive projection algorithm (SPA), the competitive adaptive reweighting algorithm (CARS), the genetic algorithm (GA), principal component analysis (PCA), random frog (RF), and Monte Carlo-uninformative variable elimination (MC-UVE) [57,58,59,60,61,62,63].

The goal of SDA is to improve classification accuracy by selecting the most relevant variables while reducing unnecessary dimensions. This helps to reduce the risk of overfitting and improve the generalization ability of the model. The SPA is a forward variable selection algorithm that eliminates redundant information in the original spectral matrix and minimizes the covariance of the variables in the spectrum [64]. CARS is a variable selection algorithm based on PLS and the Darwinian evolutionary principle of “survival of the fittest”, which filters the wavelengths by the size of absolute regression coefficients and excludes the variable bands with small weights [65]. The GA is an optimization algorithm that simulates the biological evolution process and is applied to solve complex optimization problems. Through constant selection, crossover, and mutation operations, the GA can search for combinations of feature bands with high adaptation, thus realizing the extraction of feature bands of spectral data [66]. PCA is a commonly used dimensionality reduction technique, which transforms the original data into a new set of orthogonal variables called principal components by linear transformation [67]. In spectral data feature band extraction, PCA can be employed to find the principal components that contribute most to the variability of the data and use them as feature bands. The key to the RF is continuous iteration, where a subset of features is gradually improved through natural selection and randomness operations to find the optimal combination of feature bands for classification, regression, or other data analysis tasks. The MC-UVE method utilizes Monte Carlo sampling methods to estimate the informativeness of individual bands in spectral data, which helps to identify bands that are informative for a specific task, and then the uninformative variables are eliminated to extract the final set of feature bands [68]. In Table 3, the characteristics and advantages and disadvantages of each feature extraction method are listed.

#### 3.1.3. Model Building

Spectral data modeling typically includes categorical modeling and regression modeling. Both classification modeling and regression modeling use statistical and machine-learning techniques to process spectral data for different purposes. Classification modeling is used to classify data into different categories and can be applied in the qualitative analysis of fresh tea leaf quality testing. Regression modeling is used to predict continuous output values, which can be applied in the quantitative analysis of fresh tea leaf quality testing. The methods for classification modeling are the Random Forest Classifier (RF), the K Nearest Neighbor Classifier (KNN), the Linear Discriminant Classifier (LDC), Support Vector Machines (SVMs), Extreme Learning Machines (ELMs), and the Naive Bayes Classifier (NB) [69,70,71,72,73]. Methods for regression modeling are Partial Least Squares Regression (PLSR), Multiple Linear Regression (MLR), Support Vector Regression (SVR), Extreme Learning Machine Regression (ELMR), Gaussian Process Regression (GPR), Stochastic Gradient Boosting (SGB), Kernel-based Extreme Learning Machines (KELM)s, and Random Forest Regression (RFR) [74,75,76,77,78].

The RF classifier is used to classify by integrating multiple decision tree models by voting or averaging. The KNN classifier makes classification decisions based on the neighbors of the data points. It is based on the assumption that the training samples that are close to a particular data point have similar category labels. Therefore, the KNN classifier decides the category of a new data point by summing the category labels of the K nearest neighbors weighted according to the distance [79]. The main goal of the LDC is to maximize the separation between different categories by maximizing the variance between categories and minimizing the variance within categories [80]. This makes it perform well in many classification problems, especially when the separation between categories is high. However, a limitation of the LDC is that it assumes that the data follow a multivariate normal distribution and are not applicable to nonlinear problems. For nonlinear problems, it is often necessary to use other classification methods such as SVM. The basic idea of SVM is to map the sample feature data into an n-dimensional space, where the size of n depends on the kernel function and the number of sample feature dimensions, and then construct the optimal classification hyperplane in the space [69]. A Naive Bayes Classifier uses Bayes’ theorem to estimate the posterior probability of each category for a given feature case and then selects the category with the highest posterior probability as the final classification result [81,82]. ELM is a fast and simple machine learning algorithm that achieves classification or regression tasks by randomly initializing the weights of hidden layer neurons and then training a linear output layer.

PLSR is particularly suitable for high-dimensional datasets and situations where multicollinearity problems exist. It reduces the dimensionality of the data by finding the combination of independent variables that has the highest correlation with the dependent variable, which better captures the structure of the data and builds the regression model [74,83]. MLR is a statistical method widely employed to build regression models to analyze and predict the relationship between the dependent variable and one or more independent variables. SVMR maximizes the interval between the training samples and the hyperplane by finding the optimal hyperplane in the feature space for the prediction of continuous target variables [75,84]. ELMR achieves better performance with single training by random initialization and fixing the input layer weights. KELM is an extension of the traditional ELM that introduces the kernel trick, which enables the ELM to handle nonlinear problems. GPR is a nonparametric model that utilizes a Gaussian process prior to regression analysis of input data. SGB works by integrating multiple decision trees, each trained based on a randomly selected subset of data and a subset of features, and finally voting or averaging to obtain a combined result. RFR regresses by constructing multiple decision trees and averaging them [78]. In Table 4, this paper organizes the characteristics and advantages and disadvantages of each classification model and regression model.

#### 3.1.4. Model Evaluation

The common evaluation criteria of model prediction performance are the prediction set correlation coefficient (*R*_P_), the correction set correlation coefficient (*R*_C_), the coefficient of determination (*R*^2^), prediction standard deviation (*RMSEP*), correction standard deviation (*RESEC*), and residual prediction deviation (*RPD*). The *R*_P_ is a measure of the correlation between the model’s predictions on the prediction set and the actual observations. The correlation coefficient can take values between −1 and 1, with closer to 1 indicating that the model’s predictions are more correlated with the actual values. In some fields, a correlation coefficient of 0.7 or higher may be considered good predictive performance. In practice, it is usually desirable to be close to 1. The *R*_C_ is a measure of the correlation between the model’s predictions on the correction set and the actual observations. Again, closer to 1 indicates better performance. However, an *R*_C_ that is too high may show signs of overfitting. In general, an *R*_C_ in the range of 0.7 to 0.9 may be a more appropriate range [85]. The *R*^2^ is a measure of how well the model fits the observed data. It takes a value between 0 and 1 and indicates the proportion of variance of the target variable that is explained by the model. The closer the value is to 1, the better the model fits the observed data and is able to explain more of the variance. In some fields, a value above 0.7 may be considered a better fit. Higher values are required for applications where high precision is required. *RMSEP* is a measure of how discrete the model’s prediction error is over the prediction set. It is usually asserted that the smaller this value is, the better, indicating that the model’s predictions are more stable. The *RESEC* is a measure of how discrete the model’s prediction error is on the calibration set [86]. Again, it is desired that this value be as small as possible. Residual prediction bias indicates how much the model’s predictions in the prediction set deviate from the actual observations. A smaller bias indicates that the model is more accurate.

### 3.2. Image Information Parsing

Hyperspectral image information-parsing methods include region of interest selection, image correction, dimensionality reduction, and modeling. In the study of HSI features, it is usually necessary to select the region of interest (ROI) on the leaves of fresh tea. The selection of ROI can help to reduce the dimensionality of the data, reduce the amount of computation, and focus on a specific region for detailed analysis. Black and white correction of raw images is required to eliminate noise interference and other light source interference in the camera [87]. HSI has high dimensionality and redundant data, resulting in a time-consuming computational process. There is an urgent need for dimensionality reduction processing of hyperspectral data. The methods of dimensionality reduction processing mainly include feature selection and feature extraction. Feature selection is feature band selection [88]. In order to extract the spatial texture features of the image, feature extraction of the hyperspectral image is also required. Texture feature extraction methods include the Gray-Level Co-occurrence Matrix (GLCM), the Gray-Level Difference Matrix (GLDM), the Autocorrelation Function (AF), the Local Binary Pattern (LBP), and the Wavelet transform (WT) [85,86,87]. The GLCM is a statistical tool used to describe the texture of an image. It calculates the gray-level symbiosis between pixels in an image, including information such as the angle, the distance, and gray-level differences. The GLDM is used to measure the differences between gray levels in an image. The AF measures the correlation of gray values between pixels in an image. The LBP is a nonparametric method used for the analysis of image texture. It encodes image texture features by comparing the gray values of a pixel with its neighboring pixels and then LBP histograms or other statistical information can be computed. The WT can be used to capture multi-scale texture information in hyperspectral images. Image spatial texture feature extraction can capture the detailed information in the image, which helps to identify and distinguish different textures and improve the performance of image analysis and classification. After the image dimensionality reduction process, it then needs to be modeled and analyzed. The modeling method of image information is similar to Section 3.1.3 and will not be repeated here.

### 3.3. Information Analysis for Fusion of Image and Spectral

Fusion is the fitting of an image’s spatial and spectral reflectance features into a single image. Thus, hyperspectral images integrate spectral and spatial texture features to optimize predictive capabilities. Typically, the fusion process can be performed at different levels, which can be categorized as signal level, pixel level, feature level, and decision level. Among them, signal-level image fusion is a problem of optimal concentration or distribution detection of signals and has the highest time and space requirements for alignment. Pixel-level fusion needs to process a large amount of data, which takes a relatively long time to process, is easily affected by noise, and cannot process data in real time. Decision-level fusion is the involvement of feature extraction of image data and some auxiliary information. This valuable information is combined to obtain a comprehensive decision-making result to improve recognition and interpretation. Feature-level fusion is used to extract the original information from the sensors, and then the feature information is comprehensively analyzed and processed, which can retain more original information [89]. Constructing a model after fusing features is similar to Section 3.1.3.

## 4. Application of Spectroscopic Techniques in Tea Fresh Leaf Quality Testing

### 4.1. Application of Hyperspectral Reflectance Information in Fresh Tea Leaf Quality Testing

#### 4.1.1. Quantitative Analysis Applications

Based on hyperspectral reflectance information, many researchers have quantified the physicochemical constituents such as tea polyphenols, anthocyanins, carotenoids, and catechins of tea fresh leaves to evaluate the quality of tea fresh leaves. Zhang et al. selected SG, MA, and FTIR preprocessing methods for comparative analysis [75]. The PCA method was used to extract the characteristic bands. The estimation model of the relationship between spectral reflectance and tea polyphenol content of tea fresh leaves was established using MLR, ALR, and OLS. Among them, the least squares model had the highest accuracy, and the correlation coefficient of the prediction set was 0.99. It indicated that the prediction value of the tea polyphenol content in the test samples had a small error in the measured value, and it could be realized to estimate the tea polyphenol content of tea fresh leaves on-line by using hyperspectral technology. Anthocyanins are important chemical components of tea, which have a significant impact on the color, flavor, antioxidant properties, and medicinal value of tea. Therefore, the detection of anthocyanin content in tea fresh leaves is critical for assessing the quality and value of tea. Dai et al. applied four different pre-processing methods to eliminate the effects of unfavorable factors [76]. PLS models were established using the processed data. For total anthocyanins, the PLS model with MSC-S-G-FD treatment had the best Rp and RPD values and the lowest RMSEP, showing excellent predictive performance. Sonobe et al. and Wang et al. used the PROSPECT-D model and 2-Der-PLSR inversion to estimate the carotenoid content in tea fresh leaf blades, respectively [9,90]. The results showed that HSI combined with the variable selection method can be used as a fast and accurate method to predict carotenoid content. Kang et al. determined EC, EGC, ECG, and EGCG of catechins in green tea new shoots using hyperspectral imaging [40]. The PLSR model was used, and with few exceptions, hyperspectral reflectance explained more than 79% of each catechin in the new shoots. The moisture content of tea is an important indicator for the quality testing of fresh tea leaves and has a significant impact on both the quality and shelf life of tea. Dai et al. utilized four different algorithms (SG, MSC, SNV, and OSC) to preprocess the raw data, and used stepwise regression analysis to extract characteristic wavelengths from the preprocessed data. MLR and PLSR were used to establish the quantitative analysis model of the water content of tea fresh leaves [41]. The best prediction model was the SG-OSC-SW-PLSR model, and the correlation coefficients of the model correction set, cross-validation set, and prediction set were 0.8977, 0.8342, and 0.7749, respectively, and the minimum root-mean-square errors were 0.0091, 0.0311, and 0.0371, respectively. Both Wang et al. and Mao et al. used the SPA and competitive adaptive reweighted sampling selected feature wavelengths to establish a water content regression model [42,43]. The coefficients of determination of the models were all above 0.90, which can be used to evaluate the freshness of tea leaves and provide a basis for acquisition and tea withering. Sun et al. quantitatively assessed the water content of fresh tea leaves [91]. The most effective wavelengths were first extracted using four feature selection algorithms, SPA, CARS, SPA-sr, and CARS-sr. On this basis, a spectrum-based prediction model was established by using MLR after processing 20 different combinations of algorithms. The prediction coefficient of determination of the combined algorithms of SG-MSC and CARS-sr was 0.8631, and the *RMSE*_P_ = 0.0163. The visualized distribution map of the tea leaves was able to more intuitively and comprehensively evaluate the water content of the tea leaves in each image element, which provided a new method for plant irrigation evaluation. It provides a new method for plant irrigation evaluation. It can be seen that hyperspectral technology can effectively realize the detection of water content in tea fresh leaves.

In addition, HSI data are widely used for the determination of nitrogen content and chlorophyll content of tea fresh leaves, which can provide a reference for the growth and fine management of tea plants. Nitrogen plays a pivotal role in the operation of tea plantations and has an important impact on the growth, productivity, and nutritional status of tea trees. Cao et al. proposed a method for estimating nitrogen content in tea tree fields based on the combination of a multispectral imaging system and hyperspectral data [92]. Firstly, 28 wavelengths were selected from hyperspectral data combined with 27 multispectral indices as raw data through competitive adaptive reweighted sampling. Subsequently, five variables were selected by variable combination. The results showed that the multispectral and hyperspectral data combined with SVR could effectively monitor soil nitrogen levels under field conditions, with *R*^2^ and RMSE of 0.9186 and 0.0560, respectively. Wang et al. proposed the use of SNV to preprocess hyperspectral data of mature leaves of tea trees with different nitrogen applications [52]. PLSR was utilized to predict the nitrogen content. The results showed that the diagnostic accuracy of the LS-SVM model for different nitrogen applications and nitrogen status reached 82% and 92%, respectively, with a good prediction effect. Wang et al. proposed to estimate the nitrogen content by using wavelet coefficients extracted from the CWT technique with different decomposition layers of the CWT. Finally, the CWT (lscale)-VCPA method established the best model performance, and the *R*^2^ of the model was 0.95 [53]. The accuracy was improved by 11% compared with the traditional spectral processing method. In situ determination of chlorophyll-b content as a marker for evaluating light stress and response to environmental changes in tea trees can be used to improve tea tree management. Sonobe et al. tested the performance of four machine learning algorithms, RF, SVM, Deep Belief Networks, and KELM, in evaluating tea data under different shade treatments [93]. The RMSE of KELM was 8.94 ± 3.05, showing the best performance. These results suggest that combining hyperspectral reflectance and KELM has the potential to track changes in the chlorophyll content of shaded tea leaves. Mao et al. determined the corresponding leaf physicochemical parameters and pre-processed the raw hyperspectral data collected using MSC, FD, and S-G algorithms [54]. After that, UVE and SPA were used to screen the pre-processed hyperspectral data for characteristic bands. Finally, CNN, SVM, and PLS were utilized to establish a quantitative prediction model for SPAD content. The best prediction model had an *R*^2^ of 0.730.

The above study shows that for quantitative analysis of HSI reflectance data in fresh tea leaves, the commonly used data preprocessing methods are FD, SD, and SG smoothing, the feature selection is commonly used in CARS and SPA, and the models are PLSR and SVM. However, when measuring different indexes, it is necessary to screen out specific data preprocessing methods and estimation models in combination with the actual situation in order to ensure that rapid detection is realized.

#### 4.1.2. Qualitative Analysis Applications

Qualitative studies on tea fresh leaves based on hyperspectral reflectance information have varietal classification and quality identification. Spectral information helps to capture small differences between varieties, thus giving unique spectral fingerprints to different tea varieties. Yan et al. used MSC and SNV for spectral preprocessing. The improved BP neural network, traditional BP neural network, and SVM fresh tea variety identification models were constructed. The results showed that the SVM model had the highest recognition accuracy of 96% [94]. Since different degrees of withering lead to changes in chemical composition and organizational structure in tea, these changes can be reflected in spectral data. Therefore, spectral information can help to realize the recognition of the degree of withering of tea leaves. Tu et al. collected hyperspectral data from the canopy of tea trees and classified tea varieties according to the spectral characteristics of the tea canopy [95]. Using appropriate spectral preprocessing methods, the overall accuracy of support vector machines for tea variety classification can reach more than 95%.

High-grade tea leaves have a high content of nutrients and low-grade tea leaves have relatively low content. Spectral analysis can be used to assess the quality and grade of tea by determining the content and proportion of chemical components in tea. Wang et al. combined hyperspectral technology with MBKA-Net for overall quality identification of tea leaves at different picking periods [17]. Firstly, the spectral information of six different tea-picking periods was obtained. Secondly, the MBKA method was proposed to realize the classification of tea leaves in different harvesting periods by effectively mining spectral features through multi-scale adaptive extraction. Ultimately, MBKA-Net obtained 96.18% correctness, 97.14% precision, and 97.18% recall. The study shows that the use of the variable screening method can effectively reduce the redundancy of hyperspectral information, simplify the model, and improve the model discrimination precision.

### 4.2. Application of Image and Spectral Information Fusion for Tea Fresh Leaf Quality Detection

HSI can provide detailed information on the surface microstructure and texture characteristics of tea leaves, but it has not been applied alone in the analysis of tea fresh leaf quality. It is often combined with hyperspectral reflectance information, and by fusing these two types of information, a more comprehensive and diverse set of tea leaf characteristics can be obtained. It is often applied for the qualitative analysis of tea leaves, including disease identification and variety classification.

Tea leaves usually have unique surface texture characteristics, and the change in hyperspectral image information after disease can distinguish healthy tea leaves from diseased ones and determine whether they are diseased or not. Lu et al. used hyperspectral images to identify white star disease and anthracnose in tea [96]. Preprocessing was first performed to select the best feature wavelengths for the spectral data using SPA. The diseases were then classified for prediction using SVM and ELM. The results showed that the prediction accuracy of the ELM model was higher than SVM with different kernel functions (RBF, Sigmod, and polynomial) in each disease category, and the recognition rate reached 90%. Yuan et al. proposed a new method for detecting anthracnose in tea trees based on hyperspectral imaging [97]. Two new disease indices, the tea anthracnose ratio index and the tea anthracnose normalized index, were first established based on sensitive bands. Based on the optimized spectral feature set, a disease scab detection strategy combining unsupervised classification and adaptive two-dimensional thresholding was proposed. The results showed that the overall accuracy of disease scab identification was 98% at the leaf level and 94% at the pixel level. Zhao proposed a multi-step plant adversity identification method based on HSI and CWT [98]. It was used to classify tea green leafhopper, anthracnose, and sunburn for anomaly detection. The method achieved an overall accuracy (OA) of 90.26~90.69%, with anthracnose having the highest OA (94.12~94.28%), followed by tea green leafhopper (93.99~94.20%), and sunburn having the lowest OA (82.50~83.91%).

Yan et al. used the fusion of image and spectral features as a tool for the recognition of Longjing fresh tea varieties [94]. The improved BP network was used to show the best performance, with a recognition accuracy of up to 100%, which was better than the results of analyzing with spectral features or images alone. Ning et al. used the data from the fusion of spectral and texture feature values as the input values of the LDA, SVM, and ELM models to establish a shriveling degree discriminative model [99]. When the fused data of combined spectral and textural eigenvalues were used as model inputs, the model was better than the model built based on a single eigenvalue. The overall discrimination rate reached 94.64%. The above studies have shown that the establishment of a characterization model for the integration of information is an important tool for the future use of hyperspectral “map-integrated” characterization.

### 4.3. Application of Other Spectroscopic Techniques in the Quality Testing of Fresh Tea Leaves

NIRS is widely used in quantitative and qualitative analyses of fresh tea leaves because of its sophisticated data processing methods, high accuracy, and reliability. In recent years, the effectiveness and accuracy of near-infrared spectroscopy have been fully verified in the detection of water content, catechins, caffeine, and other chemicals in tea, as well as the identification of tea varieties and the identification of tea quality. MIRS has a wide range of applications in chemical analysis and materials research, but relatively few applications in food and agriculture. Some studies have applied mid-infrared spectroscopy for the detection of dry matter, catechins, and caffeine content in tea, as well as the identification of tea varieties and the geographical origin of tea. However, due to the shallow penetration depth of the mid-infrared band, most of the studies on tea quality detection have been conducted in the near-infrared band. Compared with infrared spectroscopy, RS has the advantages of a wider determination range, convenient spectral analysis, favorable determination of aqueous solution, and simple preparation and processing of specimens. It is used to detect the carotenoid and chlorophyll content of fresh tea leaves. THz was characterized by low photon energy and good penetrability, and thus was used to detect the presence of tea stems, insects, and other foreign objects in tea. In recent years, FS has been widely used in the fields of tea grade evaluation, species differentiation, and heavy metal detection. Using FS at low concentrations, the fluorescence intensity of the solution is proportional to the concentration of the fluorescent substance. Therefore, FS was often used to detect the content of specific elements and important active ingredients in tea fresh leaves. This section summarizes the qualitative and quantitative studies of NIRS, MIRS, THz, RS, and FS in the quality detection of tea fresh leaves. It mainly includes variety identification, quality grading, disease discrimination, and the detection of tea polyphenols and other components’ content, as shown in Table 5.

## 5. Discussion

Based on the above literature, our discussion on the application of spectroscopic techniques in tea leaves mainly includes the rapid determination and prediction of tea leaf quality components such as tea polyphenols, carotenoids, and anthocyanins. We also included the classification of tea tree varieties, quality grading and quality identification of tea leaves, and the identification of tea tree pests and diseases. According to Table 6, we can see that in spectral data preprocessing, scholars mostly use SG, MSC, and SNV to smooth and correct spectral reflectance. In feature extraction, CARS and SPA are used extensively to reduce the dimensionality of spectral data for selecting effective wavelengths. Among the 21 papers listed in this paper applying hyperspectral analysis of fresh tea leaves, SG appeared nine times, MSC appeared nine times, and SNV appeared nine times. For feature extraction methods, CARS and SPA appeared six and seven times, respectively. The regression model PLSR is the most applied with a total of 10 occurrences. SVM in the classification model appeared a total of five times. Moreover, according to the final better results, PLSR, MLR, and SVM models were often used in quantitative analyses to predict the content of inbuilt components of tea fresh leaves, with the overall study showing that PLSR usually had better performances. In qualitative analyses, SVM models were mostly applied to classify and diagnose, which resulted in better discriminatory performance [76]. This may be due to the influence of light and the texture of the tea leaves themselves when collecting hyperspectral data of tea leaves. The SG and SNV can correct and eliminate this effect to some extent. Compared to spectral reflectance features, image features have not attracted much interest in fresh tea leaf quality assessment. This may be due to the fact that the information obtained when using only images to characterize the quality of tea leaves is similar to that of RGB images, whereas the cost of obtaining spectral images is much higher than that of obtaining RGB images. However, the information obtained from RGB images is limited, and scholars often fuse images with spectral data to analyze the quality of fresh tea leaves. The fused data show great feasibility in the quality assessment of tea fresh leaf quality due to the acquisition of more features, which improves the accuracy of quality assessment prediction. This is especially true for the assessment of the presence of diseases in tea leaves. Spectral reflectance features characterize the internal information of the material, which makes it possible to diagnose the disease in the early stages of the disease in tea leaves. Images are used as supplementary information to provide additional features for the pre-diagnosis of diseases, thus improving the disease diagnosis rate. After obtaining the phenotypic texture and color characteristics of tea leaves using images, an SVM or linear discriminant model was constructed to diagnose the disease by combining spectral reflectance. Generally, spectra can better characterize the component properties related to the quality of tea fresh leaves and characterize the internal properties of the lesions. Combined with image characterization of visible features such as color, damage, and texture, spectral techniques show great potential in non-destructive testing of tea fresh leaf quality.

Interestingly, based on this literature, we found that research scholars are not uniform or do not follow a certain method for selecting the region of interest (ROI) to obtain it. When doing quantitative analyses, some authors chose to use the whole leaf area as ROI, while some researchers avoided the main leaf veins to select ROI [41,42,76]. Since the ROI selection methods are different, the reflectance data obtained are different, which may also lead to inconsistent performance and bias in the final regression model. Of course, when performing qualitative analyses such as disease discrimination, scholars usually adopted semantic segmentation to separate the diseased region and used the diseased region as the ROI [11,96,97]. Simultaneously, a healthy part was selected as the ROI in order to obtain the reflectance data of the healthy and diseased regions. However, hyperspectral reflectance data are being used for non-destructive testing precisely because of their ability to reflect changes in the internal composition of tea leaves. The diseased area is segmented from the image as ROI when the leaf has already undergone qualitative changes visible to the naked eye, whereas the part of the leaf that is manually judged to be healthy may have changed in its internal composition. Such a result of ROI selection may also be the reason for inaccurate final classification results.

Table 7, Table 8, Table 9, Table 10 and Table 11 show the literature we have compiled on the application of NIRS, MIRS, THz, RS, and FS in tea fresh leaves. It is not difficult to find that NIRS is more widely used compared to several other spectrometers. This may be due to the fact that the band of NIRS is in the range of 780–2500 because the characteristic bands for observing and analyzing the intrinsic components of tea leaves such as tea polyphenols or caffeine are in the range of this band according to the results of existing literature. For several processing methods of spectral data, SNV in preprocessing was the most used with a total of 11 occurrences. PCA and PLSR were more frequently used for the screening and modeling of the characteristic bands, and according to the better results obtained, there was no one model that was universal. The preferred data processing methods chosen for different component quantitative analyses were inconsistent.

Comparing the application of HSI with NIRS, MIRS, THz, RS, and FS in tea leaves, it can be found that although HSI can acquire reflectance information and spatial image information at the same time, the commonly used HSI band is often in the range of 400–1100. However, HSI with a wider range of wavebands is particularly costly, which makes it difficult to be widely used. Although water content and nitrogen can be screened out in the 400–1100 band and some of the built-in components of tea leaves can also be screened out in the band, similar to caffeine, gallic acid, etc., whose absorption peaks are in the 2000 band, they cannot be analyzed or the results of the analysis are poor [100,102,105]. In this regard, subsequent studies could move toward the simultaneous use of HSI and other spectrometers to obtain more comprehensive spectral information on tea leaves, and thus accurately analyze the quality of tea leaves. It is interesting to note that the number of sample sets for quantitative or qualitative analyses of tea leaves is usually between 100 and 300 [52,93]. Since spectral information is usually analyzed in conjunction with physicochemical measurements, the workload involved in obtaining samples is very high, which explains the small number of sample sets. However, due to this, it tends to make the final model suffer from overfitting and poor generalization. When dealing with spectral data, how to balance the spectral information signal-to-noise ratio is also a key factor in the subsequent construction of a stable and accurate model when using smoothing, correction, and other means. At the same time, when screening the feature band dimensionality reduction, determining how to preserve the complete information as much as possible and reduce the dimensionality of the operation is also particularly important. Only after dealing with these steps can we construct a stable and accurate model for tea leaf quality analysis.

## 6. Conclusions and Prospects

This review focuses on summarizing the principles of hyperspectral imaging technology and the progress of analytical methods and applications in the quality testing of fresh tea leaves. It also briefly introduces the principles and applications of infrared and Raman spectroscopic techniques in tea quality testing. According to the previous research results of scholars, hyperspectral imaging technology and infrared spectroscopic technology have been proven to be effective tools for detecting the quality of fresh tea leaves. Compared with traditional testing methods, they are fast, highly accurate, and non-destructive, and do not require chemical reagents. The application of hyperspectral imaging technology, infrared, and other spectroscopic techniques can be used to reliably and conveniently detect the water content and quality material content components of tea leaves, thus promoting the classification of tea raw materials and assisting in the harvesting of tea leaves. But, at the same time, based on the discussion section, there are some challenges in the application of spectroscopic technology for the quality detection of tea fresh leaves:

(1) First of all, due to the chromaticity and luminosity of the capture ability, field use of spectroscopy to collect samples reflectance, by the light conditions, will affect the final test results. At the same time, in determining how to detect the quality composition content of tea fresh leaves in the tree, there are also challenges of how to select the region of interest, obtain a more consistent reflectance of the sample, and then build a stable estimation model.

(2) Secondly, the visualization and prediction technique of hyperspectral imaging provides great convenience for the detection of tea fresh leaves quality, but its high cost, large amount of imaging data, and high redundancy usually require data preprocessing by extracting the feature wavelengths through a variety of effective algorithms for dimensionality reduction, as well as building a robust calibration model for extracting the depth features. Spectral techniques such as infrared and other spectral techniques are unable to obtain image phenotypic information, meaning some information is missing. It is especially important to obtain multi-spectral images of the characteristic bands of tea leaf quality substances and reduce the amount of data without losing the characteristic information of tea leaf quality substances.

(3) Finally, after constructing the quality classification model of tea leaves and the regression of component content detection, determining how to ensure the stability of the model and the subsequent generalization performance and reduce the data running memory are also important issues in spectral technology in the quality detection of tea leaves.

## Figures and Tables

**Table 1:** Comparative analysis table of spectroscopic techniques.

Spectral Technology	Wavelength (nm)	Technical Principle	Benefits	Shortcomings
NIRS	780–2500	multiple- and combined-frequency absorption of vibrations of hydrogen-containing groups X-H (X = C, N, O) [24].	high penetration depth, weak background signal interference, high spatial, and temporal resolution [25].	spectral data processing is complex and susceptibleto moisture interference [26].
MIRS	2500–25,000	absorption of functional groups in molecules that exhibit violent fundamental frequency vibrations in the mid-infrared band [27].	high absorption intensity, high sensitivity, no sample pretreatment required.	shallow penetration depth, susceptible to moisture interference.
THz	30,000–3,000,000	absorption of molecular vibrations and rotations in the terahertz band [28].	low photon energy, good penetration, wide frequency range, and high characterization capability.	time-consuming and expensive equipment [29].
RS	/	molecular vibration information is obtained by utilizing the frequency shift and intensity change of scattered light when the sample interacts with the laser light source [30].	efficient, non-destructive and moisture free.	susceptible to fluorescence, high background signal interference, weak signal [31].
FS	200–800	characterization of fluorescence and its intensity based on the phenomenon of photoluminescence of a substance.	high sensitivity, selectivity and ease of use [32].	not widely enough applied, environmentally sensitive [33].

**Table 2:** Comparison table of different pretreatment methods.

Preprocessing	Methodologies	Specificities	Advantages	Disadvantages
normalization	MMN	linear scale	simple calculation	sensitivity to outliers
	VN	resizing vectors	maintaining spectral features	dependent on the selected spectral range
baseline correction	MSC	detection and correction of multiple scattering signals in spectra	eliminating the effect of multiple scattering on spectral data	computationally complex
	SNV	linear transformation	data standardized and easily interpretable	not applicable to non-normal distributions
	DT	eliminating trend	reducing the interference of trends in analysis	information loss
	OSC	orthogonal transform	elimination of cross-interference	higher real-time requirements
	MA	calculation of the average value	trend identification, noise reduction	produce lagged effect
noise reduction	SG	polynomial fitting	excellent fitting effect	computationally complex
	FD	calculating the rate of change	highlighting trends and changes in data	increased noise in the data
	SD	calculating curvature	highlighting curvature and variation in data	enhanced noise sensitivity
	FT	frequency and time domain transformation	ability to handle cyclical data	computationally complex
	WT	wavelet functions converted to different scales	capable of handling non-stationary and non-linear signals	complexity of processing

**Table 3:** Comparison table of feature extraction methods.

Method	Specificities	Advantages	Disadvantages
SDA	stepwise selection and exclusion of variables	reduced data dimensions	data sensitivity
SPA	continuous-projection iterative computation	elimination of redundant information	noise sensitivity
CARS	dynamically adjusting feature weights	enhances image contrast and detail	sensitivity to noise and artifacts
GA	simulation of biological evolutionary processes	For high-dimensional data	higher computational costs, results dependent on parameterization
PCA	linear transformation	reduced data dimensions	loss of partial detail information
RF	simulating a frog jumping randomly to find an optimal solution	reduced computational complexity and risk of overfitting	unstable results
MC-UVE	simulation of Monte Carlo Sampling	no a priori information required	noise sensitivity

**Table 4:** Comparison table between each classification model and regression model.

Types	Method	Specificities	Advantages	Disadvantages
classification modeling	LD	finding linear decision boundaries	effective dimensionality reduction and categorization of data	sensitivity to outliers
	KNN	voting mechanism based on neighboring samples	for multi-category and non-linear problems	noise sensitivity
	RF	integration based on multiple decision trees	high accuracy and overfitting resistance	high memory and computing resource usage
	SVM	maximum margin criterion	ideal for handling high-dimensional data	computationally complex
	ELM	single hidden layer feed-forward neural network	fast training speed	handling nonlinear problems poorly
	NB	based on bayes theorem	simple and fast calculation	assumptions of independence of characteristics may not be realistic
regression Modeling	PLSR	minimizing the covariance	reducing dimensionality and multicollinearity	easily overfitted and sensitive to noise
	MLR	minimize the residual sum of squares	simple, highly interpretable	easily influenced by collinearity
	SVMR	maximum margin criterion	suitable for handling high-dimensional data	computationally complex
	ELM	single hidden layer feed-forward neural network	fast training speeds	handling nonlinear problems poorly
	KELM	single-layer neural networks combined with kernel tricks	efficient handling of non-linear problems	computationally complex
	GPR	based on Bayesian theory and statistical learning theory	suitable for dealing with high-dimensional data, nonlinear problems	computationally complex
	SGB	Integration based on several decision trees	efficient handling of large-scale data	noise sensitivity
	RFR	Integration based on multiple decision trees	high robustness	noise sensitivity

**Table 5:** Qualitative and quantitative studies of spectroscopic techniques in tea fresh leaf quality.

Spectroscopy	Quantitative Analysis	Qualitative Analysis
NIRS	moisture content [100,101], catechin, caffeine [102,103], theanine [104], nitrogen content [105], tea polyphenol [106], flavonoids [107], EGCG [108], Heavy metals [109].	Tea varieties [110], Tea quality grade [111,112], Tea maturity [113], Traceability of Tea Raw Materials [114], diseases [115], Tea tree growing environment [116].
MIRS	Dry Matter of Tea [117], Tea polyphenols, flavonoids [118]	Tea varieties identification [106,119]
THz	Tea tree cold injury detection [120].	Separation of tea leaves from foreign matter [121], Determination of the degree of oxidation of tea leaves [122].
RS	Carotenoid measurement [123,124], Chlorophyll measurement [125]	Quality Identification [126], Anthracnose Identification [127]
FS	Chlorophyll measurement [128]	Pesticide Residue Determination [129], Diagnosis of leaf spot disease [130].

**Table 6:** Application of HSI analysis in the study of the quality of fresh tea leaves.

Appliance	Pre-Process	Feature Extraction	Modeling	Best Result	Reference
Estimation of tea polyphenols	SG, FT, Polynomial smoothing, Neighbor average method, FD, SD	PCA	LSR, MLR, Polynomial regression	Neighbor average method-FD-PCA-LSR *R*_c_ = 0.99	[75]
Detection of anthocyanin content	MSC, SNV, SG, FD	CARS, VCPA, VCPA-IRIV	PLSR, SVR	MSC-SG-FD-VCPA-SVR *R*_c_ = 0.96	[76]
Prediction of chlorophylls and carotenoids content	MSC, SNV, FD	Second derivative and regression coefficient	PLSR	SNV-PLSR *R*_p_ = 0.96, *R*_p_ = 0.93	[9]
Detection of chlorophylls	Splice correction	Vegetation index	PROSPECT–D	Splice correction-Vegetation index-PROSPECT–D *R*^2^ = 0.83	[90]
Estimating the catechin concentrations	/	/	PLSR, Mutual prediction	PLSR *R*^2^ = 0.87	[40]
Estimation of water content	SG, MSC, SNV	SR	MLR, PLSR	SG-OSC-SW-PLSR *R*_c_ = 0.83	[41]
Prediction of tea polyphenols,	SG, MSC, FD	CARS, SPA, UVE	SVM, PLSR, RF	MSC-FD-SG-CARS-PLSR *R*^2^ = 0.91	[42]
Estimation of crude fiber contents	/	SPA, CARS	PLSR, MLR	SPA-MLR, *R*^2^ = 0.84	[43]
Estimation of water content	SG, MSC, OSC	SPA, CARS, SPA-SR, CARS-SR	MLR	SG-MSC-CARS-SR-MLR *R*^2^ = 0.86	[91]
Detection of nitrogen content	SNV	Vegetation index, VCPA, CARS	PLSR, SVM, RF	SNV-CARS-SVMR *R*^2^ = 0.91	[92]
Prediction of nitrogen content	MSC, SNV, FD, SD	/	PLSR, PLS-DA, LS-VM	SNV-PLSR *R*_c_ = 0.92	[52]
Estimation of nitrogen content	SG, Detrending, FD, MSC, SNV, CWT	SPA, CARS, VCPA	PLSR	CWT-VCPA-PLSR *R*^2^ = 0.95	[53]
Detection of chlorophyll content	FD	/	RF, SVM, DBN, KELM	KELM *RMSE* = 8.94 ± 3.05	[93]
Detection of REC	MSC, SG, FD	SPA, UVE	PLSR, SVMR, CNN	MSC-FD-SG-UVE-SVMR *R*^2^ = 0.80	[54]
Longjing fresh tea Variety identification	MSC, SNV, MSC+SNV	vegetation index, PCA	SVM, BP neural network	MSC+SNV-PCA-BP neural network Recognition accuracy = 98%	[94]
Identification of tea variety	MNF	PCA, ICA	MLC, MDC, ANN, SVM	MNF-SVM-PCA accuracy = 95%	[95]
Identification of tea quality	SNV, SG	/	MBKA-Net	SNV-MBKA-Net accuracy = 96.18%	[11]
Identification of white star disease	SG, SNV, SD, Semantic segmentation	SPA	PLS-DA, SVM, ELM	SG-SPA-ELM accuracy = 95.77%	[96]
Detection of anthracnose	color image extraction ROI	vegetation index	ISODATA, 2D thresholding	ISODATA Kappa = 0.91	[97]
Detection of anthracnose	extraction ROI, Continuum removal analysis, CWA	vegetation index	SVM, FLDA, RF	CWA- vegetation index- FLDA accuracy = 94.28%	[98]
Discriminant of withering quality	/	SPA, GLCM, PCA	LDA, SVM, ELM, PLS	PCA-LDA accuracy = 94.64%	[99]

**Table 7:** Application of NIRS analysis in the study of quality of fresh tea leaves.

Appliance	Pre-Process	Feature Extraction	Modeling	Beat Result	Reference
Detection of Water content	SNV, Noise reduction, Normalization	RF, PCA, Pearson correlation analysis	SVR	RF-Pearson correlation analysis-SVR *R*_p_ = 0.99	[100]
Detection of catechin, caffeine	SG, SNV, MSC	CARS-SPA	MLR, LDA	SG-CARS-SPA-MLR *R*_p_ = 0.97	[102]
Determination of tea polyphenols	SG, SNV, Baseline	CARS, SPA, RF	PLS, MLR, LS-SVM	SNV-SPA-LS-SVM *R*_p_ = 0.98	[103]
Detection of nitrogen content	FD, External parameter orthogonalization	SPA, Ordered prediction selection, VCPA-IRIV	PLSR	EPO-VCPA-IRIV-PLSR *R*_p_ = 0.97	[105]
Estimation of total polyphenols	SNV, MSC, FD, SD	/	PLSR	MSC-PLSR *R*^2^ = 0.93	[106]
Monitoring of flavonoid content	Remove noise and baseline, MA, SG, SNV, MSC, FD, SD	/	PLSR	SG-SD-PLSR *R*_p_ = 0.95	[107]
Prediction of EGCG	SG, SNV, VN, MSC, FD	CARS, RF	PLSR, LS-SVR	CARS-LS-SVR *R*_p_ = 0.98	[108]
Detection of heavy metals	/	correlation-based feature selection	PLS, RBFNN	CFS-PLS-RBFNN *R*_p_ = 0.94	[109]
Identification of tea varieties	MSC	CARS, SWR	GRNN, PNN	MSC-CARS-SWR-PNN Accuracy = 100%	[110]
Prediction of tea quality grade	SNV, SD, FD, SD, MSC	si-PLS, GA, PCA	BP-ANN	SNV-SD-si-PLS-GA-PCA-BP-ANN *R*_p_ = 0.99	[112]
Discrimination of tea maturity	FD, SD, Mean centering, SNV, MSC, SG	PCA	BPNN, GS-SVM, PSO-SVM	SG-PCA-PSO-SVM Accuracy = 98.92%	[113]
Traceability of Tea Raw Materials	Smoothing, MSC, FD, SD	/	PLS	MSC-PLS *R*^2^ = 0.82	[114]
Discrimination of diseases	MSC, SNV, SG, KND, FD, SD	/	DPLS, DA	MSC-FD-SG-DA Accuracy = 100%	[115]
Identification of tea growing environment	Norris filter, SG, MSC, FD, Mean	/	SMLR, PCR, Si-PLS	Mean-Si-PLS *R*_c_ = 0.96	[116].

**Table 8:** Application of MIRS analysis in the study of quality of fresh tea leaves.

Appliance	Pre-Process	Feature Extraction	Modeling	Beat Result	Reference
Determination of dry matter content	Smoothing, MSC, SNV	KPCA, WPT–SA	LS-SVM, PLS	SNV-WPT-LS-SVM *R*_p_ = 0.96	[117]
Determination of polyphenols and flavonoids	/	PCA	PLS	PCA-PLS *R* = 0.98	[118]
Detection of tea stalk and insect foreign bodies	/	/	KNN	KNN Accuracy = 100%	[119]

**Table 9:** Application of THz analysis in the study of the quality of fresh tea leaves.

Appliance	Pre-Process	Feature Extraction	Modeling	Beat Result	Reference
Degrees of oxidation	/	PCA	Hierarchical cluster analysis	PCA-HCA	[120]
Detection of tea stalk and insect foreign bodies	/	/	KNN	KNN Accuracy = 100%	[121]
Assessment of cold injury	/	/	two-dimensional correlation spectroscopy-PLSR, average intensity-PLSR	2DCOS-PLSR *R* = 0.91	[122]

**Table 10:** Application of RS analysis in the study of the quality of fresh tea leaves.

Appliance	Pre-Process	Feature Extraction	Modeling	Beat Result	Reference
Detection of carotenoid content	Smooting, Normalization, MSC, Baseling, WT	SPA	PLSR	WT-SPA-PLS *R*_p_ = 0.87	[124]
Detection of photosynthetic pigments	MSC, WT, SNV, RCF, airPLS	CARS	PLSR	RCF-CARS-PLSR *R*_p_ = 0.89	[125]
Identification of tea Quality	Smooting, Normalization	PCA	LDA	Smooting-Normalization-PCA-LDA Accuracy = 100%	[126]
Anthracnose Identification	Baseline correction	PCA	/	Baseline correction-PCA Accuracy = 95%	[127]

**Table 11:** Application of FS analysis in the study of the quality of fresh tea leaves.

Appliance	Pre-Process	Feature Extraction	Modeling	Beat Result	Reference
Detection of chlorophyll content	SG	SPA, UVE	PLSR, BiPLS	SG-SPA-BiPLS *R*_p_ = 0.96	[128]
Determination of Pesticide Residue	Black and white correction	PCA	Spectral angle mappe	Black and white correction-PCA-SAM Accuracy = 100%	[129]
Diagnosis of leaf spot disease	SG	PCA	PLS-DA, SVM, LDA	SG-PCA-LDA Accuracy = 98.9%	[130]

## Data Availability

Data is contained within the article.
